# Transposable elements and the evolutionary origins of C_4_ gene expression: The story of carbonic anhydrases in *Flaveria*

**DOI:** 10.1093/plphys/kiag742

**Published:** 2026-10-06

**Authors:** Matheus E Bianconi

**Affiliations:** Assistant Features Editor, Plant Physiology, American Society of Plant Biologists, Rockville, MD, United States; Laboratoire de Recherche en Sciences Végétales, CNRS, UPS, Toulouse INP, Université de Toulouse, Castanet-Tolosan, France

C_4_ photosynthesis is a CO_2_-concentrating mechanism that enhances photosynthetic efficiency in warm, dry, and open habitats. The C_4_ mechanism relies on a biochemical cycle that operates across two adjacent cell types, namely mesophyll (M) and bundle sheath (BS) cells. Atmospheric CO_2_ that enters the leaf is converted to bicarbonate (HCO_3_^−^) in M cells, where it is initially fixed into a C_4_ acid that diffuses to BS cells, which is the site of Rubisco (ribulose-1,5-bisphosphate carboxylase/oxygenase) and Calvin-Benson-Bassham cycle reactions in C_4_ plants. In BS cells, the decarboxylation of the C_4_ acid creates a high-CO_2_ environment that saturates Rubisco, suppressing its oxygenase activity and preventing carbon loss via photorespiration. As a result, C_4_ plants can sustain high carbon assimilation rates under CO_2_-limiting conditions, and this largely explains the ecological success of numerous C_4_ lineages in tropical and subtropical grasslands and savannas (Sage et al 2012).

All enzymes with a role in C_4_ biochemistry were already present in the C_3_ ancestors but with nonphotosynthetic functions (Aubry et al 2011). The transition to a C_4_ metabolism therefore depended on spatial and quantitative changes in gene expression and protein accumulation, as well as on changes in protein catalytic properties. While the evolution of C_4_ protein sequences has been more widely studied (e.g. Christin et al 2007), the regulatory mechanisms at the DNA and protein levels controlling C_4_ enzyme expression remain largely unknown, although much progress has been made over the past 20 years (reviewed in Hibberd and Covshoff 2010; Ermarkova et al 2020).

Cis-regulatory elements controlling M-specific expression of genes encoding central C_4_ enzymes such as carbonic anhydrase (CA), phosphoenolpyruvate carboxylase (PEPC), and pyruvate orthophosphate dikinase have been identified in several C_4_ species (Hibberd and Covshoff 2010). In the genus *Flaveria* (Asteraceae), which includes species with photosynthetic phenotypes ranging from C_3_ to C_4_, a cis-regulatory element detected in C_4_ species (mesophyll expression module 1 [MEM1]) was sufficient and necessary for the accumulation of PEPC transcripts in M cells (Gowik et al 2004). An enhancer sequence involved in M-specific expression of CA was later identified also in C_4_  *Flaveria* (Gowik et al 2017). Notably, a segment of this sequence was highly similar to MEM1, and for this reason was named MEM1-like. However, the evolutionary origins of this enhancer, and the extent to which it also contributes to the high expression levels observed in C_4_ CA genes, remained unclear. In a recent article in *Plant Physiology*, Baros et al (2026) investigated these questions to retrace the series of evolutionary changes that led to the C_4_ pattern of expression of carbonic anhydrases in the model *Flaveria*.

Carbonic anhydrases are zinc metalloenzymes that catalyze the interconversion of CO_2_ and bicarbonate, a reaction that supports many physiological processes in plants, including pH regulation, photosynthesis, respiration, and stomatal movement. In C_4_ plants, they have the additional role of providing bicarbonate, which is fixed by PEPC in the cytosol of M cells. Three classes of CAs exist in land plants (α, β, and γ), but only βCA is abundant in leaves and accumulates in chloroplasts, mitochondria, cytosol, and the plasma membrane (DiMario et al 2017). In *Flaveria*, three paralogs of βCA have been identified in C_3_ species, with two of these (βCA1 and βCA3) being found exclusively in chloroplasts and one in the cytosol (βCA2). However, in the C_4_ species *F. bidentis*, βCA3 accumulates at high levels in the cytosol due to the lack of an N-terminal chloroplast transit peptide that is present in the βCA3 orthologs of C_3_ species (Tanz et al 2009).

In this new study, Baros et al (2026) sequenced transcripts and upstream genomic regions of βCA3 genes from a wider phylogenetic sampling of *Flaveria* encompassing C_3_, C_3_-C_4_, C_4_-like, and C_4_ species to investigate the genetic changes that resulted in the co-option of βCA3 for C_4_ photosynthesis. By comparing βCA3 transcript sequences isolated from all species, they found that C_4_ and C_4_-like species actually have two βCA3 paralogs (βCA3a and βCA3b), which resulted from a gene duplication event in the most recent common ancestor of the C_4_/C_4_-like clade of *Flaveria* (Fig. 1). Although protein sequences of both paralogs show N-terminal deletions relative to the C_3_ and C_3_-C_4_ orthologs, only βCA3a lost the chloroplast transit peptide and was predicted to be confined to the cytosol, while βCA3b retained its ancestral localization within the chloroplast.

**Figure 1:**
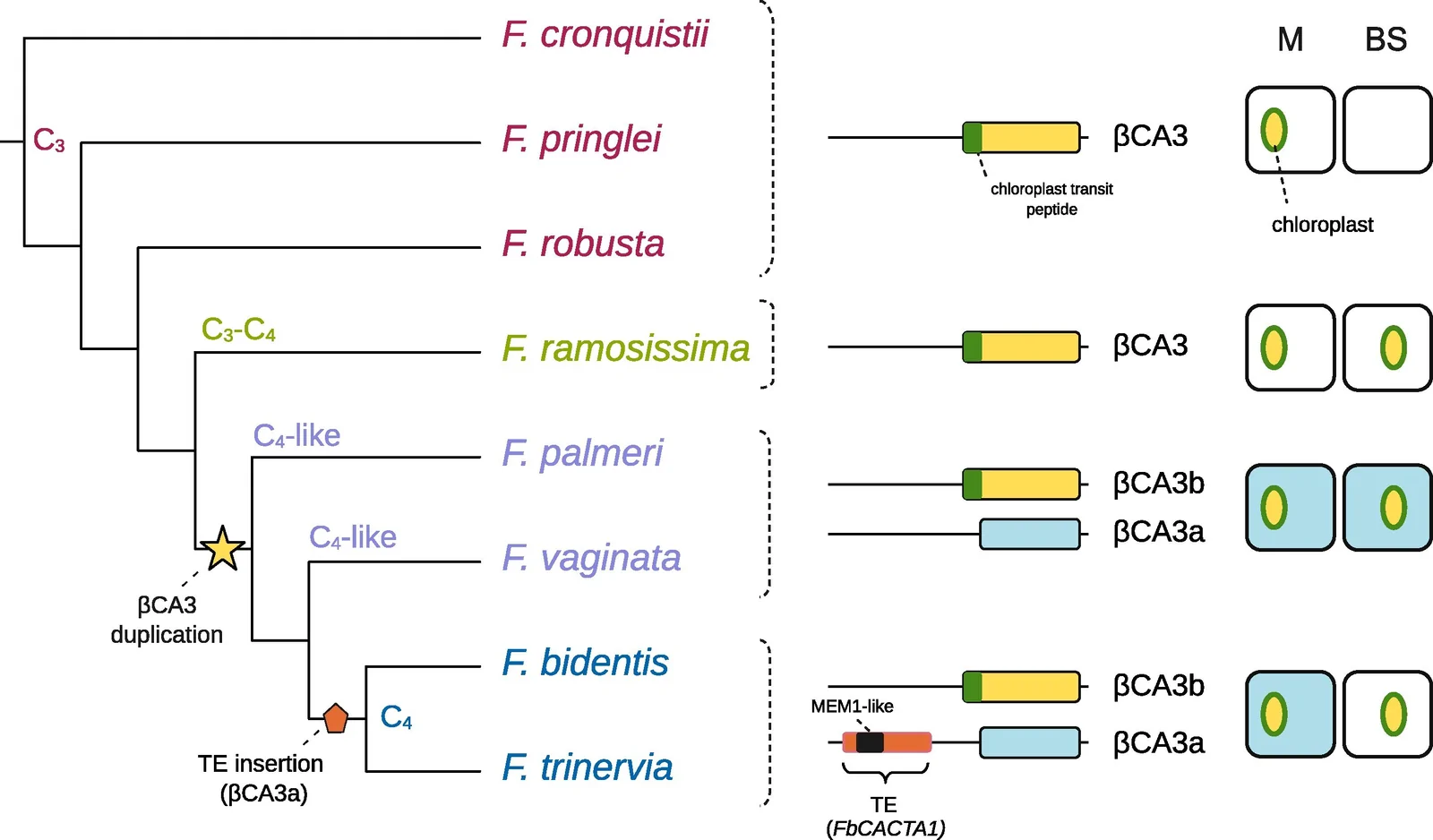
Model of C_4_ carbonic anhydrase evolution in *Flaveria*. Schematic representation of βCA3 gene sequences and their flanking regions, with the corresponding site of preferential accumulation of their encoded proteins in M or BS cells in *Flaveria* species. Two βCA3 paralogs are present in the C_4_/C_4_-like clade (βCA3a and βCA3b), and the presence of a TE of the CACTA superfamily (*FbCACTA1*) upstream to βCA3a in C_4_ species is indicated. M-specific expression of βCA3a in C_4_ species is conferred by the presence of MEM1-like upstream to the gene. Green bars on genes indicate the presence of a sequence encoding a chloroplast transit peptide. Sequence representations not in scale. Phylogenetic relationships of *Flaveria* redrawn from Baros et al (2026).

To gain insights into the regulatory mechanisms underlying the high expression of βCA3a exclusively in M cells in C_4_  *Flaveria*, Baros et al (2026) analyzed the upstream sequences of βCA, βCA3a, and βCA3b across C_4_ and non-C_4_ species. They found that the MEM1-like element that underlies the M-specific expression of βCA3 in *F. bidentis* is exclusive to the βCA3a copy. Surprisingly, the authors discovered that this element is embedded in the sequence of a transposable element (TE) that was inserted upstream to βCA3a in C_4_  *Flaveria* (Fig. 1). Further characterization of the TE sequence indicated that it belongs to the CACTA superfamily of class II TEs (DNA transposons). By inspecting the TE sequence, they noticed that it lacks the typical transposase and related protein sequences that are canonical to CACTA TEs. This observation is consistent with the process of TE domestication, in which TE insertions with adaptive consequences tend to accumulate mutations that prevent them from remobilizing in the genome, resulting in its co-option as a constitutive cis-regulatory element.

Finally, using a leaf transient transformation system that they recently developed in *F. bidentis* (Baros et al 2024), Baros et al (2026) searched for cis-regulatory sequences that are necessary and sufficient to create the high expression levels of βCA3a. By testing promoter-reporter constructs containing different portions of the upstream region of βCA3a, the authors identified a proximal promoter region that, although insufficient to explain alone the high levels of βCA3a in C_4_  *Flaveria*, may act in concert with the distal MEM1-like element to promote βCA3a expression.

The study by Baros et al (2026) reconstructs the series of genetic changes that led to the co-option of an enzyme constitutively expressed in leaves for a novel role in C_4_ biochemistry. This provides an important piece to explain the evolutionary puzzle of the C_4_ syndrome, which evolved independently in more than 60 lineages of angiosperms (Sage et al 2012). However, some questions remain to be further investigated. For example, whether homologous cis-regulatory elements can be co-opted by different C_4_ genes (as it might be the case of MEM1 in PEPC and MEM1-like in βCA) has yet to be tested. Also, could TEs play a major role by replicating such C_4_-regulatory elements throughout the genome (cf. Cao et al 2016)? Once viewed as parasitic or junk DNA that accumulates in genomes during evolution, TEs are now perceived as major drivers of evolutionary innovation (Dubin et al 2018), and they might underlie the remarkable recurrency of C_4_ evolution across angiosperm clades.

## Recent research articles in *Plant Physiology*

- Hua et al (2024) identified new candidate transcription factors controlling the expression of a C_4_ decarboxylase (NADP-ME) in sugarcane (https://doi.org/10.1093/plphys/kiae455).
- Wang et al (2026) investigated the extent to which C_4_-related features can be experimentally induced by a low CO_2_ atmosphere in non-C_4_ species of *Flaveria* (https://doi.org/10.1093/plphys/kiag089).

## Data Availability

No new data were generated or analysed in support of this News and Views article.
